# Assessing a commercially available sports drink on exogenous carbohydrate oxidation, fluid delivery and sustained exercise performance

**DOI:** 10.1186/1550-2783-11-8

**Published:** 2014-03-04

**Authors:** Justin D Roberts, Michael D Tarpey, Lindsy S Kass, Richard J Tarpey, Michael G Roberts

**Affiliations:** 1 School of Life & Medical Sciences, University of Hertfordshire, College Lane, Hatfield, Hertfordshire, UK

**Keywords:** Fructose, Maltodextrin, Exogenous carbohydrate oxidation, Fluid delivery, Performance

## Abstract

**Background:**

Whilst exogenous carbohydrate oxidation (CHO_EXO_) is influenced by mono- and disaccharide combinations, debate exists whether such beverages enhance fluid delivery and exercise performance. Therefore, this study aimed to ascertain CHO_EXO_, fluid delivery and performance times of a commercially available maltodextrin/ fructose beverage in comparison to an isocaloric maltodextrin beverage and placebo.

**Methods:**

Fourteen club level cyclists (age: 31.79 ± 10.02 years; height: 1.79 ± 0.06 m; weight: 73.69 ± 9.24 kg; VO_2max_: 60.38 ± 9.36 mL · kg^·-1^ min^-1^) performed three trials involving 2.5 hours continuous exercise at 50% maximum power output (W_max_: 176.71 ± 25.92 W) followed by a 60 km cycling performance test. Throughout each trial, athletes were randomly assigned, in a double-blind manner, either: (1) 1.1 g · min^-1^ maltodextrin + 0.6 g · min^-1^ fructose (MD + F), (2) 1.7 g · min^-1^ of maltodextrin (MD) or (3) flavoured water (P). In addition, the test beverage at 60 minutes contained 5.0 g of deuterium oxide (^2^H_2_O) to assess quantification of fluid delivery. Expired air samples were analysed for CHO_EXO_ according to the ^13^C/^12^C ratio method using gas chromatography continuous flow isotope ratio mass spectrometry.

**Results:**

Peak CHO_EXO_ was significantly greater in the final 30 minutes of submaximal exercise with MD + F and MD compared to P (1.45 ± 0.09 g · min^-1^, 1.07 ± 0.03 g · min^-1^and 0.00 ± 0.01 g · min^-1^ respectively, *P* < 0.0001), and significantly greater for MD + F compared to MD (P = 0.005). The overall appearance of ^2^H_2_O in plasma was significantly greater in both P and MD + F compared to MD (100.27 ± 3.57 ppm, 92.57 ± 2.94 ppm and 78.18 ± 4.07 ppm respectively, *P* < 0.003). There was no significant difference in fluid delivery between P and MD + F (*P* = 0.078). Performance times significantly improved with MD + F compared with both MD (by 7 min 22 s ± 1 min 56 s, or 7.2%) and P (by 6 min 35 s ± 2 min 33 s, or 6.5%, *P* < 0.05) over 60 km.

**Conclusions:**

A commercially available maltodextrin-fructose beverage improves CHO_EXO_ and fluid delivery, which may benefit individuals during sustained moderate intensity exercise. The greater CHO_EXO_ observed when consuming a maltodextrin-fructose beverage may support improved performance times.

## Introduction

It has long been established that carbohydrate (CHO) ingestion at frequent intervals, or late into submaximal aerobic exercise can maintain plasma glucose concentrations [1], and support performance through a number of mechanisms including glycogen preservation, increased total carbohydrate oxidation rates (CHO_TOT_), lowered subjective perception of fatigue and prevention of acute onset hypoglycaemia [1-3].

When exercise is of a prolonged nature (ie: >3 hours), CHO_TOT_ plays a significant role in sustaining power output (particularly if the exercise is considered strenuous). It is well established that exogenous carbohydrate oxidation rates (CHO_EXO_) may be limited at 1.0 g.min^-1^ when single sugars eg: glucose, are consumed, due to saturation of the intestinal sodium glucose cotransporter (SGLT1). The resulting contribution from endogenous carbohydrate sources to maintain CHO_TOT_ may therefore limit performance.

However, combinations of glucose, fructose and sucrose have yielded 20-55% greater CHO_EXO_ than glucose alone, through additional utilisation of a separate GLUT5 transport mechanism [4-8]. Whilst optimal CHO ingestion rates of 30–80 g.hr^-1^ have been recommended for events lasting up to 2.5 hours, no differences in CHO_EXO_ have been observed between combined and single sugar beverages at moderate CHO intakes (0.80 g.min^-1^[9]). Therefore, optimal CHO_EXO_ are likely to coincide with higher total ingestion rates of mixed sugar beverages.

Indeed, CHO_EXO_ with combined glucose and fructose beverages have been reported at 1.26 g.min^-1^ up to 1.75 g.min^-1^ with ingestion rates of 1.80 to 2.40 g.min^-1^ respectively [4]. Case study assessment of world class triathletes in our laboratory have indicated high CHO_EXO_ values of >1.75 g.min^-1^ after 3 hours of competitive paced cycling with sustained ingestion rates of 2.00 g.min^-1^ indicating potential training tolerance to carbohydrate ingestion (unpublished observations). However, such high intakes may not be practical, or indeed tolerable, by club level and recreational athletes, and may exacerbate gastrointestinal distress [10] which could be detrimental to both sustained performance and beverage delivery.

The use of maltodextrin-fructose formulas have been shown to elicit equally high CHO_EXO_[11], and may maintain gastrointestinal comfort [12]. Whilst the benefit of sports drinks on fluid delivery has been contested [13], with higher carbohydrate delivery, there is recent evidence to suggest that combined transportable sugar beverages may enhance fluid delivery [8,14-16], which may benefit the athlete when net fluid loss may impede late stage exercise performance. Less is known about commercially available sports drinks [17], particularly maltodextrin-fructose formulas, on fluid delivery.

Of key interest is the effect of sports drinks on exercise performance. The inclusion of CHO beverages has been shown to improve exercise performance and time to fatigue during relatively short laboratory [18-20] and field based assessments [21]. More recently, studies have demonstrated an effect of multiple transportable carbohydrates on sustained time trial performance [22,23] and power output [22,24]. However, this is not supported elsewhere [25], especially when commercially available carbohydrate beverages have been used [26].

With recent public interest in the accuracy of marketing claims, and whether commercially available sports drinks are indeed beneficial for performance [27,28], we were invited to undertake an independent assessment of a commercial maltodextrin/ fructose beverage (MD + F: Energy Source™, High 5 Ltd.) on total and exogenous carbohydrate oxidation, and fluid delivery in comparison to a maltodextrin only beverage (MD) and placebo (P). A further aim was to assess the influence of the three beverages on cycling performance following a period of sustained steady state exercise. It was hypothesised that the MD + F commercial formula would lead to greater exogenous oxidation and fluid delivery rates, resulting in a specific performance gains.

## Materials and methods

### Participants

Fourteen club level male cyclists were recruited for participation following power calculation assessment (G*Power3, Dusseldorf [29]). All participants had an endurance training background of at least two years, and did not suffer from diabetes or have known dysglycemia. Before undertaking the study, participants were required to provide written informed consent and satisfactorily complete a health screen questionnaire. Additionally, participants were excluded if consuming other nutritional supplements. Ethical approval for the study was provided by the University of Hertfordshire Life and Medical Sciences Ethics Committee.

### Procedures

#### Preliminary testing

At least one week prior to experimental trials, participants completed an incremental exercise test to volitional exhaustion for assessment of maximal power output (W_max_) and maximal oxygen consumption (VO_2max_). All testing was undertaken in the Human Physiology Laboratory, Division of Sport, Health and Exercise, University of Hertfordshire. Upon reporting to the laboratory, the participants’ nude body mass (Seca, model 780, Hamburg, Germany) and height were recorded. Following this, maximal tests were performed on a Computrainer (RaceMate Inc, Seattle, USA) and related Coaching Software program (Comp CS, RaceMate Inc, Seattle, USA). The Computrainer was pre-calibrated and standardised to the body mass and cycle of the participant.

Following a 10 minute standardised warm-up at 100 W, an incremental step protocol was then undertaken, with power output progressing by 30 W each 3 minutes until volitional exhaustion. Participants were fitted with an oro-nasal face mask (7920 series, Hans Rudolph Inc., Kansas City, USA) attached to a triple-V digital volume transducer. Respiratory data was recorded throughout exercise using a Metalyzer 3B system online automated gas-analyser in conjunction with Metasoft version 3 software (Cortex Biophysik, Leipzig, Germany). Heart rate (HR) was recorded continuously via radio-telemetry (Polar Electro Oy, Kempele, Finland). Ratings of perceived exertion (RPE) were collected in the final minute of each stage, using the Borg 6–20 subjective exertion scale [30]. The test concluded when participants reached volitional exhaustion or were unable to maintain the required power output.

Maximal power was calculated by adding the final completed workload to the fraction of time spent in the non-completed workload, multiplied by 30 W. Oxygen consumption (VO_2_) was defined as maximal when two of the following criteria were met: 1) a levelling off of VO_2_ with increasing workload (increase of no more than 2 ml · kgˉ^1^ · minˉ^1^); 2) attainment of maximal predicted heart rate (±10 beats.min^-1^); and 3) a respiratory exchange ratio (RER) of >1.05. The highest attained VO_2_, maintained for 20 seconds, was determined to be the VO_2max_. Participants also undertook a separate habituation trial for both steady state and performance conditions. The characteristics of the participants are shown in Table 1.

**Table 1:** Summary of participant characteristics and pre-experimental data collection

**Age (years)**	**Height (m)**	**Weight (kg)**	**VO**_**2max **_**(L.min**^**-1**^**)**	**VO**_**2max **_**(ml.kg**^**-1**^**.min**^**-1**^**)**	**W**_**max **_**(watts)**	**50% W**_**max **_**(watts)**
31.79 ± 10.02	1.79 ± 0.06	73.69 ± 9.24	4.40 ± 0.56	60.38 ± 9.36	352.64 ± 52.39	176.71 ± 25.92

Table 1 shows the key characteristics of all participants, including data for maximal power output from pre-experimental assessment. Values are presented as mean ± SD; n = 14; VO_2max_, maximal oxygen uptake; W_max_, maximal power output.

#### Experimental trials

All experimental trials were undertaken in the Human Physiology Laboratory, Division of Sport, Health and Exercise, University of Hertfordshire under controlled conditions (temperature: 22.4 ± 0.9°C; barometric pressure – range: 979–1023 mBar; and relative humidity – range: 21–56%). No differences were reported between trials (*P* > 0.05) for any of the environmental variables.

The study employed a randomised, placebo-controlled, double-blind cross over design for beverage condition. Participants were required to perform three exercise trials separated by one week, each comprising a 2.5 hour cycle at 50% W_max_ (oxidation trial), followed by a 60 km cycling test (performance trial). Trials were undertaken at the same time of day to minimise the potential for diurnal variance. Participants reported to the laboratory following a 12 hour overnight fast. Upon arrival, nude body mass was measured and participants rested for 5 minutes before baseline measurements (for expired air and blood analytes) were undertaken. All trials were performed on a pre-calibrated Computrainer (RaceMate Inc, Seattle, USA), as employed in preliminary testing.

#### Sub-maximal oxidation trial

Following a 10 minute warm up at 100 W, participants began a 2.5 hour oxidation trial at 50% W_max_. Steady state power output was based on individual quantification of W_max_ from pre-experimental assessment. Expired air samples were collected via the Douglas bag method at 30 and 60 minutes, and then 15 minute intervals thereafter, and analysed for percentage O_2_ and CO_2_, using a Servomex 1440 gas analyser (Servomex Group Ltd, Crowborough, UK). Total Douglas bag volume was measured using a dry gas meter (Harvard Apparatus, Holliston, USA). Standardised measurements for minute ventilation (V_E_, L.min^-1^), oxygen uptake (VO_2_, L.min^-1^), carbon dioxide (VCO_2_, L.min^-1^) and respiratory exchange ratio (RER) were recorded at 0, 30 and 60 minutes, and every 15 minutes thereafter during the oxidation trial.

In addition, immediately following each Douglas bag collection, duplicate 10 ml expired air samples were extracted into vacuumed Exetainer tubes (Labco Ltd, High Wycombe, UK) for the determination of expired gas ^13^C:^12^C ratio. Exetainer samples were analysed independently (Iso-Analytical Ltd., Crewe, UK) for ^13^C:^12^C ratio by gas chromatography continuous flow isotope ratio mass spectrometry (GC-IRMS, Europa Scientific 20–20 IRMS). Stable isotope measurements and indirect calorimetry were used to calculate rates of CHO_EXO_, CHO_TOT_ (total carbohydrate oxidation) and FAT_TOT_ (total fat oxidation).

At rest, and at 15 minute intervals throughout the oxidation trial, 30 μl of capillarised wholeblood was collected in heparinised tubes and frozen at -8°C for subsequent analysis of blood glucose using an Analox micro-stat PGM7 (Analox Instruments Ltd, London, UK). Telemetric HR was recorded at 15 minute intervals throughout the oxidation trial. Ratings of perceived exertion (RPE_TOTAL_ and RPE_LEGS_) using the 6–20 and 0–10 Borg scales respectively were recorded every 30 minutes during submaximal exercise. Participants also verbally completed an adapted 14 point gastrointestinal (GI) symptom assessment questionnaire [31] every 30 minutes, grading the degree of subjective discomfort on a 0–10 visual analogue scale. Particular attention was given to symptoms categorised as both ‘moderate’ (4–6) and ‘severe’ (7–10).

#### Beverage administration

In a double-blind random order manner, participants were assigned the following beverages across trials: maltodextrin only (MD), isoenergetic maltodextrin with fructose (MD + F) or aspartame sweetened, citrus flavoured water (P). All CHO beverages were supplied by High 5 Ltd., and prepared as 10% concentrated formulas in opaque drinks bottles. The test beverages provided an average CHO delivery rate of 1.7 g · min^-1^ for MD (corn-derived glucose monohydrate), and 1.1 g · min^-1^ maltodextrin with 0.6 g · min^-1^ fructose for MD + F (using corn-derived glucose monohydrate and crystalline fructose, Energy Source™, High 5 Ltd.). This was based on discussion with the manufacturer to provide a realistic, but high ingestion rate (102 g total carbohydrate per hour in line with high dosages recommended for longer duration exercise).

Beverages were administered as controlled 270 ml doses at the start of the oxidation trial and every 15 minutes (until completion of the performance trial), providing a fluid intake of 1.08 L · h^-1^. In terms of content, the test beverages per 100 g comprised: i) for MD + F – 96.7 g of total carbohydrate (of which 59.7 g from maltodextrin, 31.5 g from fructose); 0.0 g of protein and fat; and delivered 388 kcal; ii) for MD - 96.0 g of total carbohydrate (of which 90.9 g from maltodextrin, 4.0 g from fructose); 0.0 g of protein and fat; and delivered 384 kcal; and P – 0.3 g of total carbohydrate (of which 0.3 g total sugars); 0.2 g of protein and 0.0 g of fat; and delivered 10 kcal.

All CHO beverages contained 816 mg per 100 g (~35.5 mmol.L^-1^) of sodium (as tri-sodium citrate and sodium chloride). Corn-derived glucose monohydrate and crystalline fructose were used due to their naturally high ^13^C content, allowing for the quantification of CHO_EXO_. The ingested glucose and fructose were subject to elemental analyser-isotope ratio mass spectrometry (EA-IRMS; Europa Scientific 20–20) for the determination of ^13^C-enrichment (MD: -11.41 δ‰, MD + F: -11.84 δ‰ vs. Pee Dee Bellemnitella (PDB)).

#### Assessment of fluid delivery

The quantification of plasma deuterium enrichment has previously been validated for qualitative assessment of fluid delivery [8,14]. Based on both sample size power determination (G*Power 3, Dusseldorf) and cost, it was deemed that only 7 participants were required for assessment of fluid delivery. Prior to the oxidation trial, an intravenous 20 gauge cannula was inserted by a qualified phlebotomist into an antecubital vein for 7 of the participants, to allow repeated blood sampling. Sample lines were kept patent after each blood collection with a 2 ml isotonic saline flush (0.9% sodium chloride saline, Baxter, Norfolk, UK).

Participants received 5 g of deuterium oxide (^2^H_2_O, Sigma Aldrich, Dorset, UK), included in the beverage administered at 60 minutes, for assessment of fluid delivery. Blood samples were collected in 10 ml Vacutainer tubes, containing sodium fluoride/K_3_EDTA as an anticoagulant (Beckton Dickinson, Plymouth, UK), at 15 minute intervals from the 60 minute time point into the oxidation trial. Blood samples were analyzed for plasma ^2^H_2_O enrichment via equilibration (Europa 20–20 continuous-flow isotope ratio mass spectrometry) by an independent laboratory (Iso-Analytical Ltd., Crewe, UK). Indwelling cannulas were removed at the end of the oxidation trial.

#### Performance trial

Upon completion of the oxidation trial, participants performed a 60 km performance trial using the same Computrainer (RaceMate Inc, Seattle, USA). This was based on manufacturer recommendation to simulate durations encountered during sportive level events. Treatment beverages continued to be ingested every 15 minutes in the same quantity as for the oxidation trial. Participants were instructed to complete the 60 km cycle course in the fastest possible time, and were given verbal encouragement throughout the test coinciding with beverage administration.

Telemetric HR and capillarised wholebood (for glucose analysis as previously described) were assessed at 15 minute intervals. In line with laboratory safety regulations, participants were required to stop exercising if blood glucose dropped below 2.5 mmol·L^-1^. Gastrointestinal symptom assessment was undertaken every 30 minutes as previously described. Speed (km.hr^-1^), power output (W) and distance covered (km) were recorded during the performance trial at 15 minute intervals, but with an adapted monitor only permitting sight of distance covered. At the cessation of the test, participants cooled down for 5 minutes at 100 W.

#### Trial control measures

All participants were required to maintain a food and exercise diary for 7 days prior to the first exercise trial, and maintain these patterns before each subsequent trial. Participants were provided with a list of foods naturally abundant in ^13^C (CHO derived from C4 plants, e.g.: corn and sugar cane) and instructed to avoid them for the 7 days prior to the first exercise trial and for the duration of the experimental period to reduce background ^13^C from endogenous stores. Food lists also provided a number of alternative high CHO foods to prevent a reduction in CHO intake. Additionally, to reduce background interference from ^13^C-enriched glycogen stores, participants performed a 150–180 minute glycogen-depleting ride 5 days before each trial. Previous studies have employed similar interventions to limit the effects of background ^13^C-levels [5,7,8]. Participants were asked to refrain from caffeine, alcohol ingestion and intense exercise for 24 hours before each trial.

#### Calculations

Total oxidation rates:

Rates of CHO_TOT_ and FAT_TOT_ (g · min^-1^) were calculated from absolute VO_2_ and VCO_2_ (L · min^-1^) utilising the following stoichiometric equations [32], with protein oxidation during exercise assumed negligible:

$$\mathrm{CH}O_{\mathrm{TOT}}=4.585\left(\mathrm{VC}O_{2}\right)3.226\left(VO_{2}\right)$$

$$\mathrm{FA}T_{\mathrm{TOT}}=1.695\left(VO_{2}\right)‒1.701\left(\mathrm{VC}O_{2}\right)$$

Exogenous carbohydrate oxidation rates:

The rate of CHO_EXO_ (g · min^-1^) was calculated using the following formula [33]:

$$\mathrm{CHOEXO}=\mathrm{VCO}2\cdot \left(\frac{\mathrm{Exp}‒\mathrm{Ex}p_{\mathrm{BKG}}}{\mathrm{Ing}‒\mathrm{Ex}p_{\mathrm{BKG}}}\right)\cdot \left(\frac{1}{k}\right)$$

Where δExp is the ^13^C-enrichment of expired air throughout the oxidation trial, δIng is the ^13^C-enrichment of the CHO solution,$\;{\mathrm{Exp}}_{\mathrm{BKG}}$ is the ^13^C-enrichment of expired air throughout the placebo trial (P) and *k* is the CO_2_ produced via the oxidation of 1 g of glucose (*k* = 0.7467 litres of CO_2_ per gram of glucose [8]).

The ^13^C-enrichment was expressed as δ‰ difference between the ^13^C:^12^C ratio of the sample and a known laboratory reference standard (PDB) according to the following formula [34]:

$$\delta^{13}C=\left[\left(\frac{\frac{{\;}^{13}C}{{\;}^{12}C}\;\mathrm{of}\;\mathrm{sample}}{\frac{{\;}^{13}C}{{\;}^{12}C}\;\mathrm{of}\;\mathrm{standard}}\right)‒1\right]\cdot 10^{3}$$

The rate of CHO_ENDO_ was calculated by subtracting CHO_EXO_ from CHO_TOT_. Substrate oxidation was calculated over the final 90 minutes of exercise (60–150 minutes) due to the earlier capture of ^13^CO_2_ in the bicarbonate (HCO_3_ˉ) pool. It has been reported that dilution in the bicarbonate pool becomes negligible after 60 minutes of exercise, when 100% of the ^13^CO_2_ from oxidation is retrieved [8,35,36].

Carbohydrate oxidation efficiency:

Estimation of carbohydrate oxidation efficiency was determined using the following formula [7]:

$$\mathrm{CHOEXO}\;\mathrm{efficiency}=100\cdot \left(\frac{\mathrm{CHOEXO}\;}{\mathrm{CHO}\;\mathrm{ingestion}\;\mathrm{rate}}\right)$$

Statistical analyses:

Statistical analyses were performed using SPSS Statistics for Windows version 19 (SPSS, Chicago, USA). A two-way analysis of variance (ANOVA) with repeated measures design was used to assess for interaction effects between conditions, trials and over time. Where appropriate, a one-way ANOVA was used to assess for differences for relevant experimental measures (e.g.: mean CHO_EXO_) between trials only. Significant differences were assessed with a student t-test with Bonferoni post hoc adjustments. Where pertinent, pearson chi squared assessment was undertaken (e.g.: gastrointestinal responses). An alpha level of 0.05 was employed for assessment of statistical significance. All data are reported as means ± SE.

## Results

### Submaximal oxidation trial

#### Total carbohydrate oxidation

**Figure 1:**
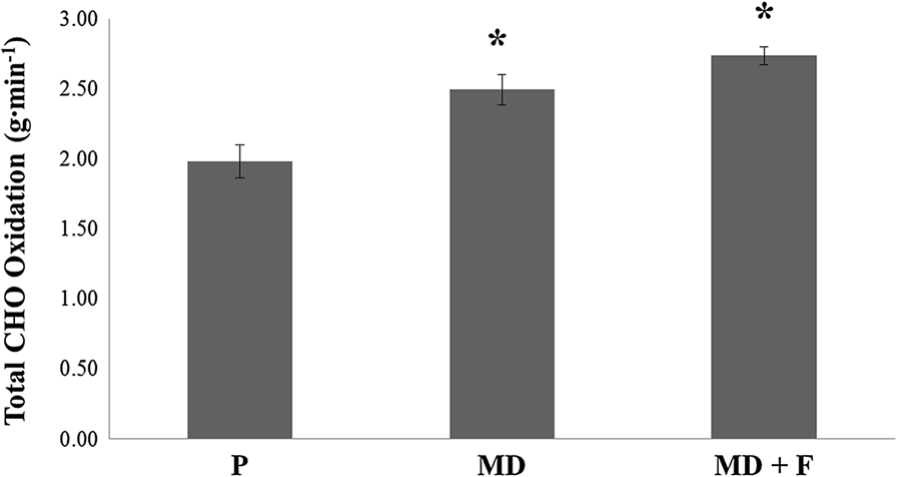
**Assessment of test beverages on mean CHO**_**TOT **_**oxidation rates between 60–150 minutes of the submaximal exercise trial.** Figure 1 demonstrates the influence of all test beverages on mean total carbohydrate oxidation rates in the final 90 minutes of the oxidation trial. Data are presented as mean ± SE; n = 14. P, Placebo; MD, maltodextrin beverage; MD + F, maltodextrin-fructose beverage; CHO_TOT_, total carbohydrate oxidation rates. *denotes significant difference (*P* < 0.001) to P.

**Figure 2:**
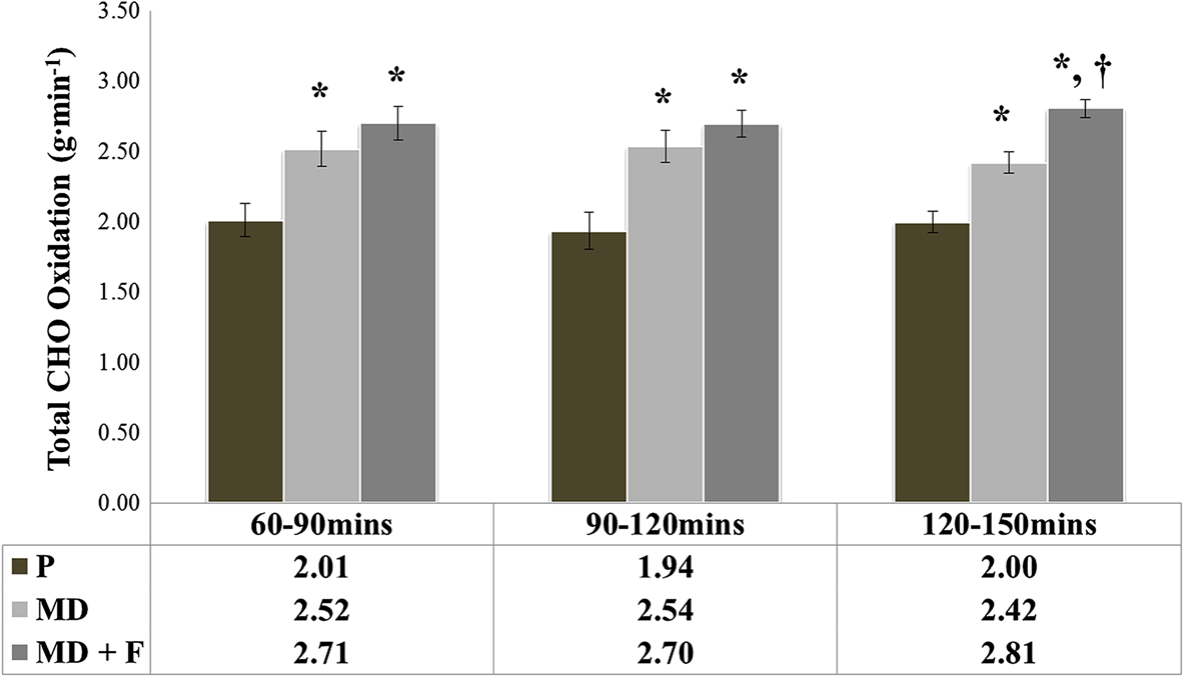
**Assessment of test beverages on mean CHO**_**TOT **_**oxidation rates at various timepoints during the submaximal exercise trial.** Figure 2 shows the difference between test beverages for total carbohydrate oxidation rates at specific 30 minute time periods in the final 90 minutes of the oxidation trial. Data are presented as mean ± SE; n = 14. P, Placebo; MD, maltodextrin beverage; MD + F, maltodextrin-fructose beverage; CHO_TOT_, total carbohydrate oxidation rates. *denotes significant difference (*P* < 0.005) to P within timepoint assessment. † denotes significant difference between MD and MD + F within timepoint assessment (*P* = 0.004).

Data for total carbohydrate oxidation rates are represented in Figures 1 and 2. During steady state aerobic exercise performed at 50% W_max_, mean CHO_TOT_ between 60–150 minutes were significantly different between treatment conditions (F = 20.601; *P* = 0.0001). Mean CHO_TOT_ were significantly greater for both MD + F and MD compared with P throughout the last 90 minutes of steady state exercise (2.74 ± 0.07 g.min^-1^ for MD + F and 2.50 ± 0.11 g.min^-1^ for MD v 1.98 ± 0.12 g.min^-1^ for P respectively; *P* = 0.0001). Mean CHO_TOT_ were not shown to be statistically different between MD + F and MD (*P* > 0.05).

When assessing specific intervals during the oxidation trial, between 60–90 minutes and 90–120 minutes of submaximal exercise performed at 50%W_max_, both carbohydrate test beverages demonstrated higher mean CHO_TOT_ compared to P (*P* < 0.005). Whilst this observation continued in the last 30 minutes of the oxidation trial, a significant difference was also found between MD + F and MD between 120–150 minutes of the test (2.81 ± 0.06 g.min^-1^ v 2.42 ± 0.10 g.min^-1^ respectively; *P* = 0.004).

#### Exogenous carbohydrate oxidation

**Figure 3:**
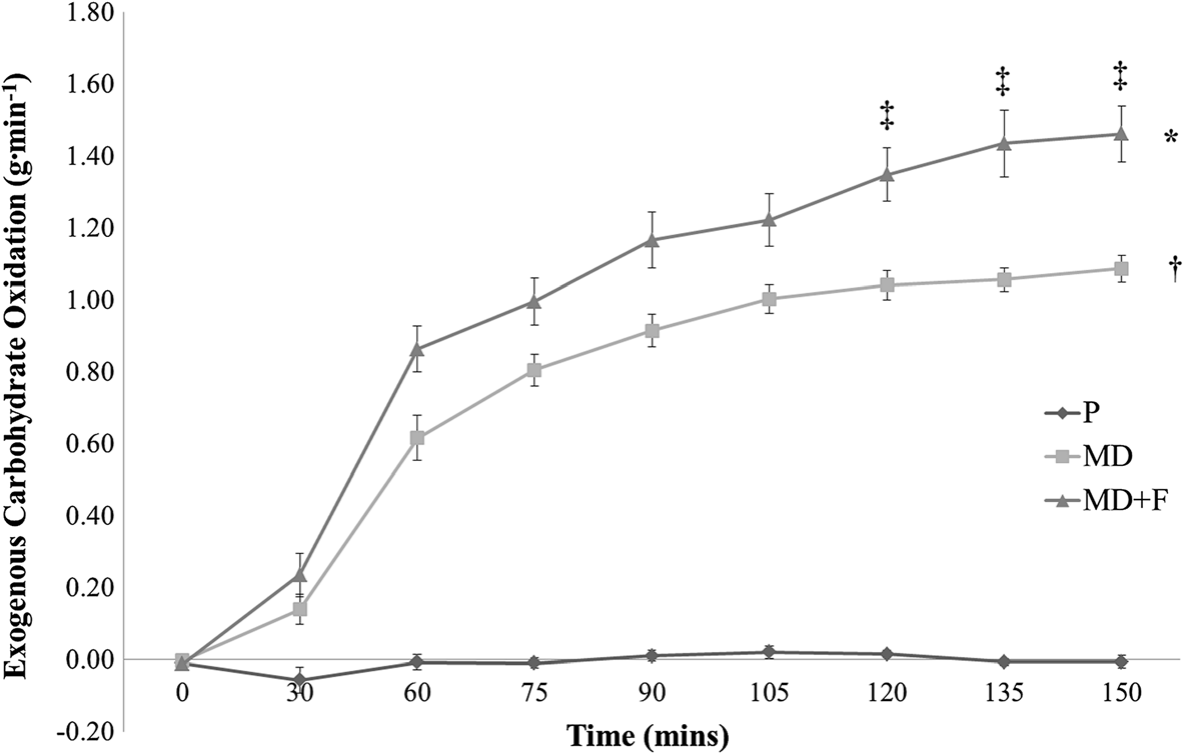
**Assessment of test beverages on exogenous CHO oxidation rates during the submaximal exercise trial.** Figure 3 demonstrates the time course effect of the test beverages on exogenous carbohydrate oxidation rates. Data are presented as mean ± SE; n = 14. P, Placebo; MD, maltodextrin beverage; MD + F, maltodextrin-fructose beverage. *denotes an overall significant difference between MD + F and P (*P* = 0.0001). † denotes an overall significant difference between MD and P (*P* = 0.0001). ‡denotes a significant difference between MD and MD + F at specific timepoint (*P* < 0.008).

Data for CHO_EXO_ are represented in Figure 3 and Table 2. A significant interaction effect was found for both time and beverage (F = 31.659; *P* = 0.0001). Whilst no differences were observed between conditions at rest (*P* > 0.05), both carbohydrate beverages displayed significantly higher CHO_EXO_ at all timepoints from 30 minutes in comparison to P (*P* < 0.0001). Mean CHO_EXO_ between 60–150 minutes was significantly different between test conditions (F = 180.077; *P* = 0.0001). Both carbohydrate beverages displayed significantly greater mean CHO_EXO_ compared with P (*P* = 0.0001). However, throughout the final 90 minutes of steady state exercise, CHO_EXO_ was significantly higher with MD + F compared with MD (1.27 ± 0.07 g.min^-1^ v 0.98 ± 0.04 g.min^-1^ respectively; *P* = 0.019). When analysed for respective 30 minute time periods, CHO_EXO_ was significantly higher for MD + F compared with MD between 90–120 minutes and 120–150 minutes only (*P* < 0.025). Peak CHO_EXO_ was significantly greater in the final 30 minutes of submaximal exercise with MD + F and MD compared to P (1.45 ± 0.09 g · min^-1^, 1.07 ± 0.03 g · min^-1^ and 0.00 ± 0.01 g · min^-1^ respectively, *P* < 0.0001), and significantly greater for MD + F compared to MD (P = 0.005).

**Table 2:** Influence of test beverages on carbohydrate and fat oxidation rates during a submaximal exercise test

		**Overall**	**Respective time period assessed**		
		**60-150 mins**	**60-90 mins**	**90-120 mins**	**120-150 mins**
CHO_ENDO_	P	1.97 ± 0.12	2.00 ± 0.12	1.92 ± 0.12	1.99 ± 0.12
(g.min^-1^)	MD	1.51 ± 0.10*	1.66 ± 0.12*	1.52 ± 0.10*	1.35 ± 0.10*
	MD + F	1.47 ± 0.07*	1.62 ± 0.08*	1.41 ± 0.07*	1.36 ± 0.07*
CHO_EXO_	P	0.00 ± 0.00	-0.00 ± 0.01	0.02 ± 0.01	-0.00 ± 0.01
(g.min^-1^)	MD	0.98 ± 0.04*	0.86 ± 0.04*	1.02 ± 0.04*	1.07 ± 0.03*
	MD + F	1.27 ± 0.07*†	1.08 ± 0.07*	1.28 ± 0.07*†	1.45 ± 0.09*†
CHO_EXO Eff_	P	0.1 ± 0.3	-0.1 ± 0.05	0.9 ± 0.8	-0.6 ± 0.8
(%)	MD	57.9 ± 2.1*	50.5 ± 2.5*	60.1 ± 2.3*	63.0 ± 1.9*
	MD + F	74.7 ± 4.4*†	63.5 ± 4.2*	75.5 ± 4.3*†	85.2 ± 5.0*†
FAT_TOT_	P	0.59 ± 0.06	0.58 ± 0.06	0.60 ± 0.06	0.58 ± 0.06
(g.min^-1^)	MD	0.41 ± 0.05*	0.42 ± 0.05*	0.41 ± 0.05*	0.41 ± 0.05*
	MD + F	0.33 ± 0.04*	0.34 ± 0.03*	0.34 ± 0.04*	0.32 ± 0.04*

Table 2 demonstrates the influence of the test beverages on endogenous and exogenous carbohydrate and fat oxidation rates during the submaximal exercise trial. Data for carbohydrate oxidation efficiency are also shown to demonstrate the progressive benefit of a combined sugar beverage overall and at 30 minute averaged timepoints. Data are presented as mean ± SE; n = 14. P, Placebo; MD, maltodextrin beverage; MD + F, maltodextrin-fructose beverage. CHO_ENDO_, endogenous carbohydrate oxidation; FAT_TOT_, total fat oxidation; CHO_EXO_, exogenous carbohydrate oxidation; CHO_EXO Eff,_ carbohydrate oxidation efficiency *denotes a significant difference (*P* < 0.038) to P within respective time period. † denotes a significant difference between MD and MD + F (*P* < 0.025) within respective time period.

Assessment of exogenous carbohydrate efficiency (CHO_EXO Eff_%) was additionally undertaken across the oxidation trial. Mean CHO_EXO Eff_% was significantly greater with MD + F and MD compared to P for all assessed time periods (*P* < 0.0001). Additionally CHO_EXO Eff_% was significantly greater with MD + F compared to MD overall (74.7 ± 4.4% v 57.9 ± 2.1% respectively; *P* = 0.019), and at respective assessed timepoints from 90 minutes (*P < 0.025*).

#### Endogenous carbohydrate oxidation

Data for mean CHO_ENDO_ are represented in Table 2. In a similar pattern to mean CHO_TOT_, a significant interaction effect was found between treatment conditions for mean CHO_ENDO_ between 60–150 minutes of the oxidation trial (F = 13.822; *P* = 0.0001). Both MD + F and MD conditions demonstrated lower mean CHO_ENDO_ during the last 90 minutes of continuous exercise compared to P (1.47 ± 0.07 g.min^-1^, 1.51 ± 0.10 g.min^-1^ and 1.97 ± 0.12 g.min^-1^ respectively; *P* < 0.004). Whilst mean CHO_ENDO_ progressively declined for each averaged 30 minute period within treatment condition, the same pattern was observed with both carbohydrate beverages demonstrating significantly lower CHO_ENDO_ in comparison to P (*P* < 0.038). No differences were observed between MD + F and MD (*P* > 0.05).

#### Total fat oxidation

Data for mean FAT_TOT_ are shown in Table 2. Over the final 90 minutes of the oxidation trial, mean FAT_TOT_ was statistically different between conditions (F = 10.494; *P* = 0.0001). Specifically, both carbohydrate beverages demonstrated lower mean FAT_TOT_ in comparison to P (*P* = 0.008). Whilst absolute values were lower for MD + F in relation to MD, mean FAT_TOT_ was not statistically different between carbohydrate beverages (0.33 ± 0.04 g.min^-1^ for MD + F v 0.41 ± 0.05 g.min^-1^ for MD, *P* > 0.05) over the final 90 minutes of the oxidation trial. The same observation was noted for all 30 minute intervals, with both carbohydrate beverages demonstrating significantly lower mean FAT_TOT_ in comparison to P only (*P* < 0.021).

Assessment of exercise intensity was deemed comparable during the oxidation trial, with no significant differences observed for mean absolute VO_2_ (L.min^-1^) and power output (W) between test conditions – see Table 3.

**Table 3:** Assessment of oxygen uptake, power output, mean heart rate, blood glucose and perceived exertion during both the oxidation and performance trials

		**Oxidation trial**	**Performance trial**
VO_2_ (L.min^-1^)	P	2.65 ± 0.07	N/A
	MD	2.69 ± 0.06	N/A
	MD + F	2.70 ± 0.09	N/A
Power (W)	P	176.3 ± 6.95	201.0 ± 22.4
	MD	175.0 ± 6.67	197.6 ± 21.6
	MD + F	174.4 ± 6.59	227.0 ± 23.2*
Heart rate (b.min^-1^)	P	128.7 ± 4.7	149.0 ± 6.3
	MD	132.4 ± 3.7	151.9 ± 6.3
	MD + F	133.1 ± 4.4^†^	160.7 ± 5.0*
Blood glucose (mmol.L^-1^)	P	3.90 ± 0.11	3.24 ± 0.25
	MD	4.77 ± 0.12^†^	4.17 ± 0.22^†^
	MD + F	4.97 ± 0.12^†^	4.18 ± 0.23^†^
RPE_TOTAL_ (6–20 scale)	P	11.9 ± 0.6	15.6 ± 0.6
	MD	12.2 ± 0.5	16.3 ± 0.5
	MD + F	11.6 ± 0.6	16.4 ± 0.7
RPE_LEGS_ (0–10 scale)	P	3.8 ± 0.4	7.1 ± 0.4
	MD	4.2 ± 0.5	7.1 ± 0.3
	MD + F	3.3 ± 0.4‡	6.9 ± 0.6

Table 3 shows the average data for key physiological, power output and subjective perception of exertion vaiables over both the oxidation and performance trials. Data are presented as mean ± SE; (n = 14 for oxidation trial; n = 6 for performance trial finishers). P, Placebo; MD, maltodextrin beverage; MD + F, maltodextrin-fructose beverage; RPE, Rating of Perceived Exertion. *denotes a significant difference to MD and P (*P < 0.03*). † denotes significant difference to P (*P* < 0.05).‡ denotes a significant difference to MD (*P* = 0.021) within trial only.

#### Fluid delivery assessment

**Figure 4:**
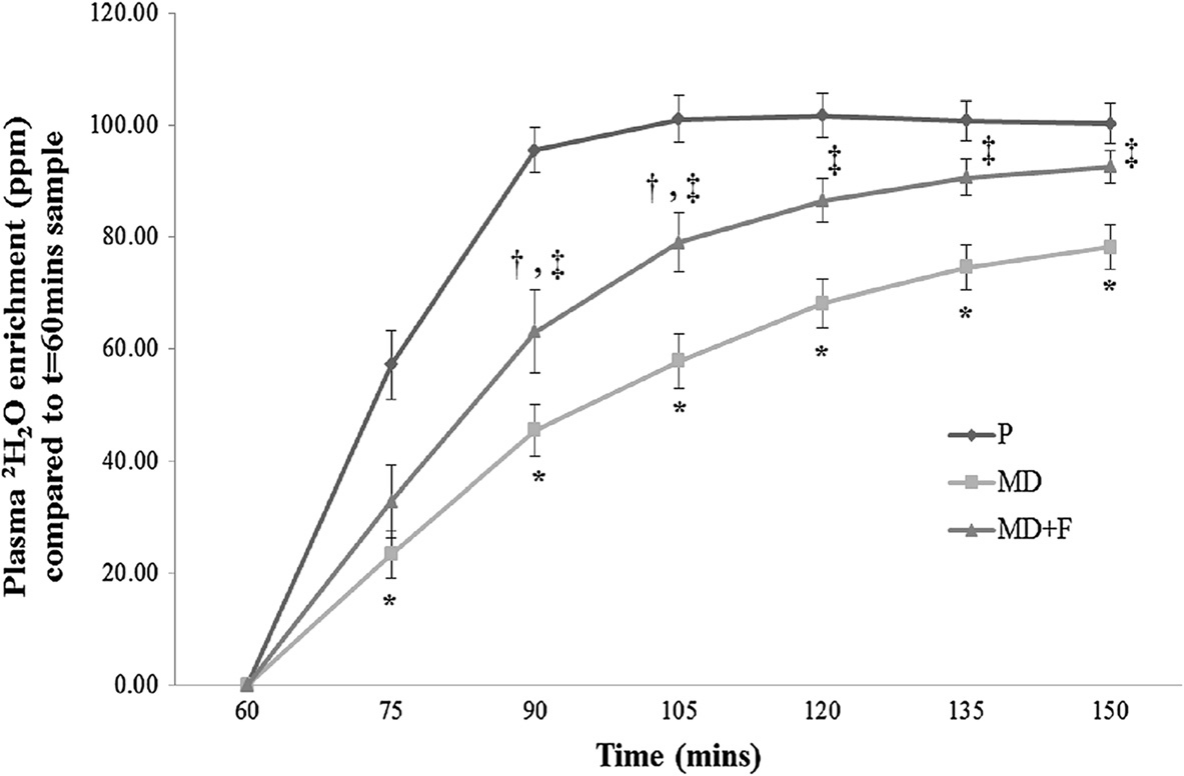
**Influence of beverage administration on plasma deuterium enrichment (ppm).** Figure 4 shows the impact of the test beverages on plasma deuterium enrichment, which was employed as a semi-quantitative method for assessing fluid delivery. Data are presented as mean ± SE; n = 7. P, Placebo; MD, maltodextrin beverage; MD + F, maltodextrin-fructose beverage. *denotes significant difference (*P* < 0.025) to P. † denotes significant difference (*P* < 0.039) to P. ‡ denotes significant difference between MD and MD + F (*P* < 0.05).

Estimation of total fluid delivery, as assessed via plasma ^2^H_2_O enrichment is demonstrated in Figure 4. As the deuterium oxide was provided within the 60 minute beverage, this timepoint was employed for baseline comparisons. The increase in plasma ^2^H_2_O enrichment from 60 minutes served to quantify total fluid delivery both within treatment condition and in comparison to P. Plasma ^2^H_2_O enrichment increased in all conditions over time (F = 55.491; *P* = 0.0001), demonstrating the greatest increase in the P condition, with a peak of 101.67 ± 3.87 ppm by 120 minutes of the oxidation trial, and thereafter plateauing with an end value of 100.27 ± 3.56 ppm.

Plasma ^2^H_2_O enrichment was significantly lower in the MD condition from 75 minutes in comparison to P (*P* < 0.025), and from 90 minutes in comparison to MD + F (*P* < 0.05). In contrast, values for plasma ^2^H_2_O enrichment were statistically lower for MD + F compared to P at the 90 and 105 minute timepoints only (*P* < 0.039). The ingestion of MD + F resulted in a peak plasma ^2^H_2_O enrichment of 92.57 ± 2.94 ppm by the end of the oxidation trial, and was comparable to values obtained for P (100.27 ± 3.56 ppm; *P* > 0.05).

### 60 km performance trial

#### Performance trial measures

**Figure 5:**
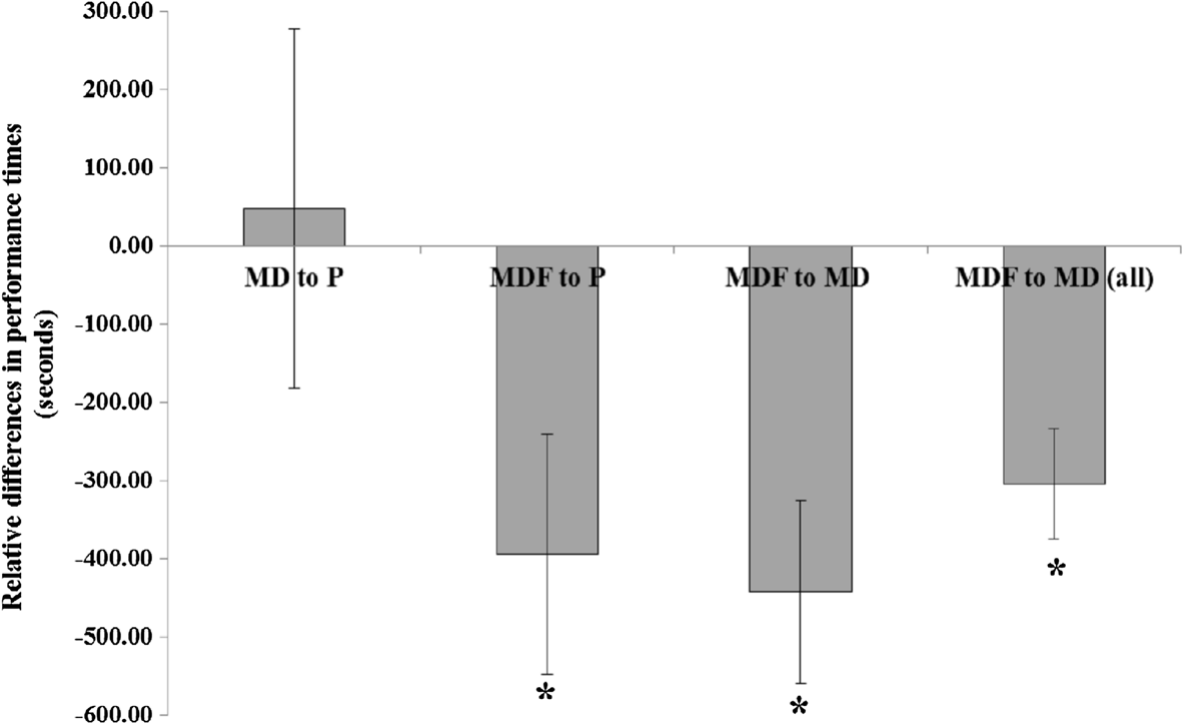
**Relative differences in 60 km performance times between beverages.** Figure 5 indicates the difference in performance times during the preloaded 60 km time trial when test beverages were compared for all finishers. The final column is included to demonstrate that all participants completed the test when consuming carbohydrate beverages. P, Placebo; MD, maltodextrin beverage; MD + F, maltodextrin-fructose beverage. Data are presented as mean ± SE; comparisons made for finishers of all trials (first three columns: n = 6) and between test beverages for all finishers (end column: n = 14) *denotes significant difference between relative beverages (*P* < 0.05).

Whilst all participants attempted the 60 km performance trial, during the P condition, 8 athletes were unable to finish demonstrating the exhaustive nature of the protocol. In contrast, all participants completed the performance trial whilst consuming both carbohydrate test beverages. Statistical analysis was therefore carried out on all finishers (n = 6) for comparison across trials. Relative differences in performance times between beverages are shown in Figure 5. Additionally, inclusion of all finishers (n = 14) for the two test beverages are shown for interest.

Performance times were significantly faster with MD + F compared with MD and P (5722.8 ± 284.1 seconds v 6165.0 ± 257.9 seconds v 6117.5 ± 358.0 seconds respectively; *P < 0.05*). In absolute terms, performance times significantly improved with MD + F compared with both MD (by 7 min 22 s ± 1 min 56 s, or 7.2%) and P (by 6 min 35 s ± 2 min 33 s, or 6.5%, *P* < 0.05) over 60 km. No difference was observed for performance times between MD and P (*P > 0.05*). The difference observed between MD + F and MD was further noted when assessment of all 14 finishers was separately undertaken (5868.36 ± 151.31 seconds for MD + F v 6217.14 ± 150.93 seconds for MD; *P* = 0.001).

**Figure 6:**
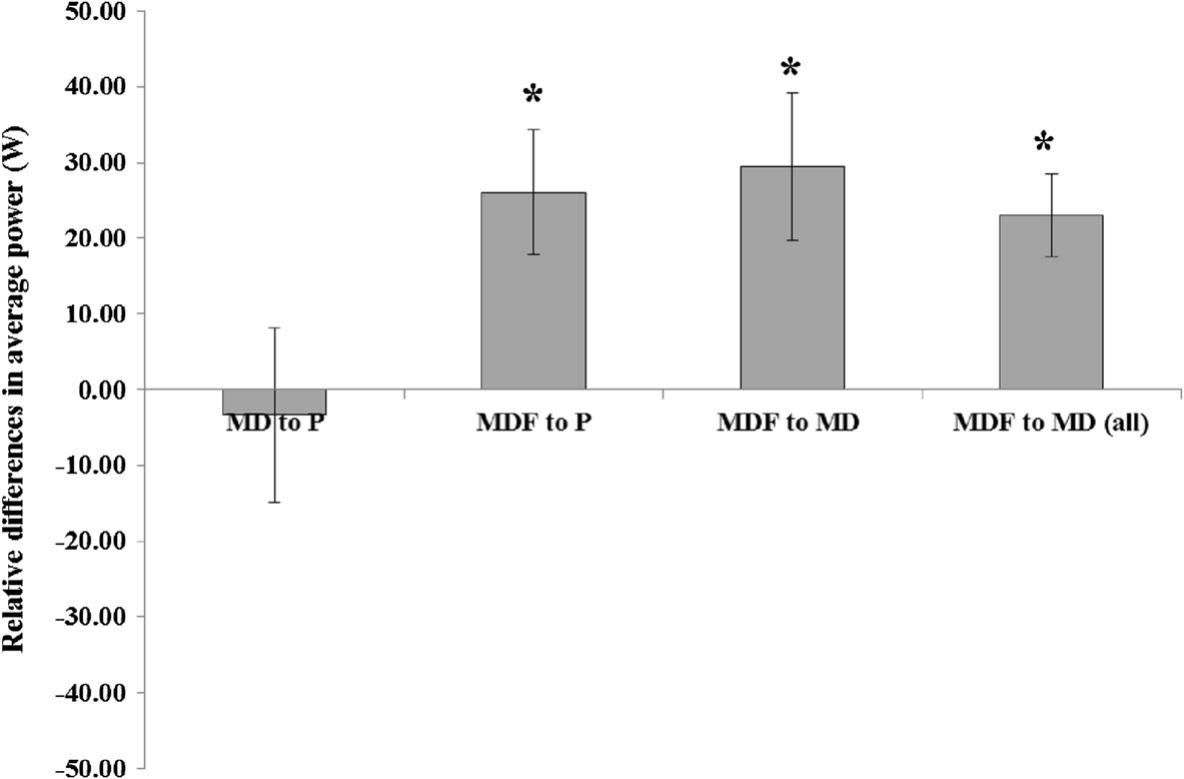
**Relative differences in average power output between beverages during the performance trial.** Figure 6 indicates the difference in average power measured in watts, during the preloaded 60 km time trial when test beverages were compared for all finishers. The final column is included to demonstrate that all participants completed the test when consuming carbohydrate beverages. P, Placebo; MD, maltodextrin beverage; MD + F, maltodextrin-fructose beverage. Data are presented as mean ± SE; comparisons made for finishers of all trials (first three columns: n = 6) and between test beverages for all finishers (end column: n = 14) * denotes significant difference between relative beverages (*P* < 0.05).

In a similar manner, relative differences in mean power output was significantly different for MD + F compared to both MD and P for the performance trial (*P < 0.03*; Figure 6). Mean power output was 14.9% greater with MD + F compared to MD (227.0 ± 23.2 W v 197.6 ± 21.6 W, *P = 0.029*), and 13.0% greater with MD + F compared to P (227.0 ± 23.2 W v 201.0 ± 22.4 W, *P = 0.025*). No difference was observed for performance times between MD and P (*P > 0.05*). The difference observed between MD + F and MD was further noted when assessment of all 14 finishers was separately undertaken (234.0 ± 12.0 W for MD + F v 204.3 ± 11.1 W for MD; *P* = 0.001).

### Other physiological and subjective measures during both trials

#### Heart rate, perceived exertion, blood glucose and gastrointestinal distress assessment

Data for mean heart rate (b.min^-1^), blood glucose and subjective perceived exertion are shown in Table 3. During the oxidation trial, mean heart rate was marginally lower with P (F = 4.059; *P* = 0.029), but only statistically different to MD + F (*P* = 0.045). However, as no differences were observed for RPE_TOT_, absolute VO_2_ or power output (*P* > 0.05) compliance to the exercise intensity was deemed appropriate. Blood glucose was significantly greater with both test beverages in comparison to P during the oxidation trial (F = 26.505; *P* = 0.0001), although no differences existed between MD and MD + F (4.77 ± 0.12 mmol.L^-1^ and 4.97 ± 0.12 mmol.L^-1^ respectively, *P* > 0.05). Mean subjective RPE_LEGS_ (using a 0–10 Borg Scale) was significantly lower for MD + F compared with MD (*P* = 0.021) over the course of the oxidation trial.

During the performance trial, greater participant effort was demonstrated via increases in mean heart rate, RPE_TOTAL_ and RPE_LEGS_ in comparison to the oxidation trial. However, as 8 athletes could not complete the performance trial for P, comparisons were made for finishers of all trials only. Mean heart rate was significantly higher with MD + F (160.7 ± 5.0 b.min^-1^) compared to both MD and P (151.9 ± 6.3 b.min^-1^ and 149.0 ± 6.3 b.min^-1^ respectively, *P < 0.03*). Mean blood glucose was similar between test beverages during the performance trial (4.18 ± 0.23 mmol.L^-1^ for MD + F and 4.17 ± 0.22 mmol.L^-1^ for MD), with both being significantly greater than P (3.24 ± 0.25 mmol.L^-1^) only (*P < 0.05*). No differences were observed between test conditions for RPE_TOTAL_ or RPE_LEGS_ during the performance trial (*P > 0.05*).

Overall responses to the gastrointestinal distress questionnaire are shown in Table 4. A higher number of significantly positive responses were noted for MD. Bloating and belching severity were considerably greater with MD (22.2% and 19.0%) compared to MD + F (<4.8%) and P (<1.6%) respectively (*P < 0.05*). Whilst responses for other symptoms were considered minor ie: <7% of all responses, it was noted that symptoms of nausea, stomach problems, and urge to vomit or defecate were observed in the MD trial.

**Table 4:** Influence of test beverages on overall gastrointestinal distress responses

**Symptom**	**P**	**MD**	**MD + F**
Urge to urinate	33 (26.2)*	17 (13.5)	19 (15.1)
Bloating severity	2 (1.6)	28 (22.2)*	6 (4.8)
Belching severity	2 (1.6)	24 (19.0)*	5 (4.0)
Nausea	1 (0.8)	8 (6.3)*	0 (0.0)
Stomach problems	1 (0.8)	7 (5.6)*	0 (0.0)
Stomach cramps	0 (0.0)	1 (0.8)	0 (0.0)
Headaches	1 (0.8)	2 (1.6)	0 (0.0)
Intestinal cramps	0 (0.0)	0 (0.0)	0 (0.0)
Stomach burning	1 (0.8)	2 (1.6)	0 (0.0)
Flatulence severity	0 (0.0)	2 (1.6)	0 (0.0)
Left & right side aches	3 (2.4)	0 (0.0)	1 (0.8)
Dizziness	8 (6.3)*	1 (0.8)	2 (1.6)
Urge to defecate	0 (0.0)	4 (3.2)*	0 (0.0)
Urge to vomit	0 (0.0)	4 (3.2)*	0 (0.0)

Table 4 shows the overall data for responses to the gastrointestinal distress questionnaire, with particular attention given to responses rated moderate to severe. Data are presented as total number of responses (rated moderate to severe) for both oxidation and performance trials. Numbers in brackets represent data expressed as a percentage of maximum number of responses. P, Placebo; MD, maltodextrin beverage; MD + F, maltodextrin-fructose beverage. *denotes a significant difference to other test conditions (*P < 0.05*).

## Discussion

The aim of this study was to carry out an independent assessment of a commercially available sports drink on carbohydrate oxidation, fluid delivery and sustained performance. Whilst previous research has indicated benefits of consuming multiple transportable carbohydrates [11,12,16,22], there is minimal research on commercial formulas demonstrating such mechanisms in line with performance gains. Additionally, there is continued interest as to whether sports drinks are indeed beneficial to recreational and club level athletes, with implications that moderately higher dosing strategies may yield effective results for longer duration events. With current dosage recommendations for events lasting longer than 2 hours being >90 g.hr^-1^[4], we were asked to investigate the potential influence of a commercial MD + F beverage provided at a relatively high carbohydrate delivery rate (102 g.hr^-1^) on club level athletes.

The main finding from the study was that a commercial MD + F beverage significantly enhanced both CHO_EXO_ and fluid delivery during steady state exercise compared to both MD and P. This resulted in an average higher power output and time to complete the subsequent 60 km time trial. The findings support previous research that combined sugar beverages provided at reasonably high concentrations (~10%) and carbohydrate delivery rates may enhance exercise performance [22,24]. This should be interpreted with a degree of caution for the end-user based on total exercise duration. For events ranging from 2 to 6 hours, such findings may be applicable. However, for shorter duration events, there is little evidence that ‘multiple transportable carbohydrates’ provide any ergogenic benefit over that of maltodextrin or glucose based beverages. Indeed, for events < 90 minutes, water only strategies may offer equally valid benefits [37]. Furthermore, as the duration exceeds 6 hours, beverage and gastrointestinal tolerance may have a negative impact on performance, indicating a potential need for lower dosing and fluid delivery strategies [10,38].

In this study, both test beverages resulted in higher CHO_TOT_ compared with P during exercise undertaken at 50% W_max_. As steady state exercise intensity was comparable across trials (for oxygen uptake, power output and perceived exertion), the use of P resulted in a higher rate of CHO_ENDO_ and FAT_TOT_, which was expected. The inclusion of the two test beverages resulted in lower CHO_ENDO_, potentially decreasing reliance on hepatic glucose utilisation, and permitting glycogen sparing, particularly in type I muscle fibres, during continuous aerobic exercise. Indeed, as the use of carbohydrate beverages has been shown to spare glycogen early into exercise [39], this may provide a subtle benefit late into exercise if CHO_TOT_ is enhanced.

Whilst CHO sparing from endogenous sources was apparent with both test beverages across all time points, it was specifically noted that CHO_TOT_ was 16.1% greater with MD + F compared to MD in the final 30 minutes of the oxidation trial. This differs from previous research utilising similar dosing strategies of fructose: maltodextrin [11], which is surprising considering CHO_EXO_ rates during the same time frame were significantly increased and comparable to values observed in the current study. As there was a progressive increase in CHO_EXO_ with MD + F throughout the oxidation trial (with mean CHO_EXO_ of 1.27 g.min^-1^ being significantly greater than MD), this implies that intestinal saturation was not a limiting factor at this dosage, as supported elsewhere [5,11]. During the MD trial, CHO_EXO_ was maintained from 90 minutes indicating potential saturation of the SGLT1 transporter mechanism. As there was no significant difference in either average CHO_EXO_ or carbohydrate oxidation efficiency between the test beverages prior to this, the use of combined sugar beverages may be more applicable for events lasting longer than 90 minutes, supporting current recommendations [4].

It should also be noted that participants in this study undertook the oxidation trial following an overnight fast. Whilst this is not normal practice for trained athletes competing, it has been shown that the influence of low dietary carbohydrate availability prior to sustained exercise has little impact on accumulated CHO_EXO_ and steady state performance [40] in the presence of CHO beverages. However, more prolonged states of starvation have been shown to reduce CHO_EXO_[41]. In the current study, participants maintained their habitual diet which was unlikely to significantly impact on CHO_EXO_.

Peak CHO_EXO_ for MD + F compared well with previous research [5,8,11], with values reaching 1.45 ± 0.09 g.min^-1^, 35.5% greater than MD, by the end of the oxidation trial. When lower ingestion rates of 0.8 g.min^-1^ have been employed to replicate practices employed by athletes (48 g.hr^-1^), peak CHO_EXO_ were not significantly different between glucose + fructose versus glucose only beverages (0.56 v 0.58 g.min^-1^ respectively, [9]). Therefore, benefits of combined sugar beverages, particularly for longer duration events, are more likely with higher ingestion rates >1.0 g.min^-1^. Interestingly, when higher ratios of fructose to maltodextrin have been employed [12], it has been suggested that peak CHO_EXO_ may occur with a 0.8 F: MD ratio compared to 0.5 or 1.25 ratios at ingestion rates of 1.8 g.min^-1^. However, as the relative concentrations of the beverages employed were >10%, CHO_TOT_ was considerably lower than the current study, and short duration performance gains observed [12] may not be replicated with longer duration events.

In the current study, the ratio of F: MD was 0.54 delivered at an ingestion rate of 1.7 g.min^-1^ (based on product analysis). This resulted in a higher CHO_TOT_ than previously observed with a 0.8 ratio [12], most likely based on higher CHO_EXO_ and lower beverage concentration, which may not have limited gastric emptying rates or intestinal beverage delivery. It is unknown whether peak CHO_EXO_ during this study would have been greater if the oxidation trial had been extended. However previous research has indicated a relative maintenance so long as ingestion rates are maintained and tolerated [42]. The ingestion of a commercially available MD + F sports drink used in this study supports the general contention that the inclusion of fructose to a glucose/maltodextrin beverage will involve both SGLT1 and GLUT5 transport mechanisms leading to an increased rate of total carbohydrate delivery across the intestinal lumen.

Although higher ingestion rates of 2.4 g.min^-1^ have been previously employed, leading to higher peak CHO_EXO_ rates of 1.75 g.min^-1^[7], it is likely that a higher beverage concentration, or total fluid consumption, would have led to progressive gastrointestinal disturbances within this cohort based on subjective reporting of drink tolerance at the end of the study. At the ingestion rates employed, it was apparent that gastrointestinal issues were less evident with MD + F compared to MD, but also that relative tolerance was being reached by the end of the performance trial. Higher ingestion rates may be better tolerated by well-trained athletes, as supported elsewhere [7] and from observations of world class triathletes in our laboratory in which peak CHO_EXO_ have exceeded 1.75 g.min^-1^ with CHO ingestion rates of 2.0 g.min^-1^. Whether this indicates a training adaptation or tolerance to beverage consumption, or full saturation of SGLT1 and GLUT5 is unknown. More likely, as trained endurance athletes are encouraged to consume high carbohydrate diets to facilitate recovery and repetitive training bouts, higher CHO_EXO_ may be the result of high carbohydrate availability, irrespective of total muscle glycogen and GLUT4 expression [40].

An important finding from the study was that plasma ^2^H_2_O enrichment was significantly enhanced with the inclusion of the MD + F formula, and statistically no different to P in the last 30 minutes of the oxidation trial. As plasma ^2^H_2_O enrichment has been used elsewhere as a semi-quantitative method [14,16], the finding indicates that MD + F did not restrict fluid delivery. However, findings for the MD beverage were significantly lower than P at all timepoints. The most likely explanation is that the ingestion of MD + F resulted in higher overall CHO_TOT_ and CHO_EXO,_ particularly in the final 30 minutes of the oxidation trial. As saturation of the SGLT1 transporter may have occurred with MD, fluid uptake across of the intestinal lumen may have been restricted. The inclusion of fructose, however, may have prevented complete intestinal SGLT1 saturation, hence allowing continued fluid uptake.

Our results are comparable to previous research [8,14,16], although plasma ^2^H_2_O enrichment values were deemed higher in the current study where an MD + F beverage was used. In previous studies, increasing beverage concentration above 6% resulted in reduced fluid delivery based on a glucose only beverage [14]. Whilst this may, in part, explain findings for the MD beverage, it would appear that the combined use of MD + F at a 10% concentration did not restrict fluid delivery. During events lasting longer than 2 hours where acute dehydration and carbohydrate depletion may limit sustained performance, the use of a commercial MD + F beverage may therefore support both high fluid delivery and CHO_EXO_ rates.

The use of combined carbohydrate beverages has been shown to enhance exercise performance [22-24]. However, several of these studies did not assess CHO_EXO_ to support conclusions, or use commercial formulas more applicable to the end user. Recent studies have indicated that running performance may not be enhanced when commercial beverages are employed [26]. In the current study, 8 participants were unable to complete the 60 km performance test, demonstrating the demanding nature of the protocol. However, data for finishers of all trials indicated that performance times and corresponding mean power outputs were significantly improved with MD + F. Mean power output was 14.9% higher during the MD + F trial compared to MD, and 13% higher compared to P.

This observation compares with previous findings [22], and may be a consequence of the higher CHO_TOT_ and CHO_EXO_ at the end of the oxidation trial with MD + F. Surprisingly mean power output was comparable between MD and P, which may indicate subjective perception of the test beverages and hence relative effort, despite being randomly assigned to trial order. As all participants were able to complete the performance trial when consuming the test beverages, this demonstrates the benefit of regularly consuming CHO during sustained exercise. However, in a similar manner, performance times and mean power output was significantly improved with MD + F compared with MD for all participants (n = 14).

Whilst RPE_TOTAL_ and RPE_LEGS_ were comparable during the performance test between conditions, it was noted that mean blood glucose was higher with both MD and MD + F compared to P. As power output was higher in the MD + F condition, this correlated with greater cardiovascular exertion despite similar perceived effort. As both test drinks were matched for electrolyte content, the buffering of endogenous acids is unlikely to be a key mechanism explaining greater power output with MD + F. Instead, higher CHO_TOT_ and potential for liver glycogen sparing with MD + F most likely explains the significant increase in performance.

It is difficult to compare data from previous research when different types of performance tests have been employed. When shorter distance preloaded time trials have been assessed, the use of glucose only beverages resulted in a dose response effect, with 60 g.hr^-1^ leading to a 10.7% increase in mean power over 20 km compared to lower dosages [43]. However, as a limiting factor for longer duration events may be CHO_EXO_, such results may not extend to longer time trials when single carbohydrate beverages are used. Furthermore, performance times during sustained endurance events, such as Ironman Triathlon, have been shown to correlate with higher total CHO intakes ranging from 90–120 g.hr^-1^[10], despite also relating to a higher incidence of gastrointestinal responses. In the current study, gastrointestinal responses did not impede performance, although it was observed that underlying responses were lower with MD + F compared to MD, similar to previous studies [5].

Where longer time trials (>100 km) have been performed (without prior steady state exercise), findings are mixed [44-46] both for low (0.62 g.min^-1^[44]) and moderate (1.10 g.min^-1^) ingestion rates [45]. As a higher ingestion rate was employed in the current study, along with greater beverage concentration, the high CHO_TOT_ and CHO_EXO_ rates observed with MD + F may explain the improved performance during a 60 km time trial in comparison to these studies. Additionally, if ergogenic effects occur following peak CHO_EXO_, then overall trials lasting <120 minutes may not be sufficient to observe performance benefits from combined sugar beverages.

## Conclusions

The use of a commercially available MD + F formula resulted in greater increases in total and exogenous carbohydrate oxidation rates during sustained steady state exercise compared to an isoenergetic MD beverage, and P. Additionally, the inclusion of fructose resulted in matched fluid delivery compared with P, and resulted in performance gains in direct comparison to MD. Athletes undertaking sustained exercise greater than 2 hours should consider strategies utilising combined carbohydrate formulas to maximise carbohydrate and fluid delivery, which may support enhanced exercise performance.

## Abbreviations

CHO: Carbohydrate; CHO_TOT_: Total carbohydrate oxidation rate (measured in g.min^-1^); CHO_EXO_: Exogenous carbohydrate oxidation rate (measured in g.min^-1^); CHO_ENDO_: Endogenous carbohydrate oxidation rate (measured in g.min^-1^); FAT_TOT_: Total fat oxidation rate (measured in g.min^-1^); GLUT5: Glucose transporter 5; MD: Isoenergetic maltodextrin formula used in the study; MD + F: Maltodextrin plus fructose formula used in the study; P: Placebo formula used in the study; RER: Respiratory exchange ratio, the ratio from dividing expired carbon dioxide with oxygen uptake; RPE: Rating of perceived exertion (subscript relates to total or leg related exertion); SGLT1: Sodium dependent glucose transporter 1; V_E_: Minute ventilation, the amount of air breathed in one minute (L.min^-1^); VO_2_: Volume of oxygen uptake (measured in L.min^-1^); VO_2max_: Maximal oxygen uptake (measured in L.min^-1^); VCO_2_: Volume of expired carbon dioxide (measured in L.min^-1^); W_max_: Maximal power output determined from an incremental protocol to volitional exhaustion (measured in W).

## Competing interests

Research funding and product supply to support this study was received from High 5 Ltd. All data was collected, analysed and reported by the investigatory team fully independently of the company.

## Authors’ contributions

All authors were involved in the study. JDR was the principal researcher, involved with liaison with the company, participant assessment, data collection, statistical analysis and manuscript generation; MDT was co-researcher involved with cohort organization, data collection and blood analyses, confirmation of statistical analyses, and manuscript editing; LSK was involved with monitoring of data collection including collation of performance data, and test beverage administration, as well as manuscript editing; RJT was involved with data collection and analysis; MGR was involved in quality control, data collection, and technical accuracy in preparation of the manuscript. All authors read and approved the final manuscript.
